# Patients with Obstructive Sleep Apnea and Cardiovascular Diseases: What, When, and Why Is Mandibular Advancement Device Treatment Required? A Short Review

**DOI:** 10.3390/jcm11226845

**Published:** 2022-11-19

**Authors:** Cindy François, Arthur Bonafé, Camille Roubille, François Roubille, Isabelle Dupuy-Bonafé, Sarah Millot

**Affiliations:** 1 Département Fonction-Dysfonction, Imagerie et Biomatériaux, Centre de Soins d’Enseignement et de Recherche Dentaires, 34080 Montpellier, France; 2 PhyMedExp, INSERM U1046, CNRS UMR 9214, University of Montpellier, 34295 Montpellier, France; 3 Département de Parodontologie, Centre de Soins d’Enseignement et de Recherche Dentaires, 34080 Montpellier, France; 4 Department of Internal Medicine, Lapeyronie Hospital, CHU Montpellier, 34090 Montpellier, France; 5 Cardiology Department, Hôpital Lapeyronie, PhyMedExp, University of Montpellier, INSERM, CNRS, CHRU, INI-CRT, 34090 Montpellier, France; 6 Département de Médecine et Chirurgie Orale, HCL, 69002 Lyon, France; 7 INSERM 1149 Faculté de Médecine Xavier Bichat, 75018 Paris, France

**Keywords:** obstructive sleep apnea, mandibular advancement device, cardiovascular disease, dental sleep medicine

## Abstract

Obstructive sleep apnea is a potentially dangerous condition with significant risks of comorbidities if left untreated. It represents a cardiovascular risk factor in the general population, and a higher prevalence is observed in patients already suffering from cardiovascular diseases. The gold standard treatment, continuous positive airway pressure, is not always accepted or tolerated. The mandibular advancement device represents an alternative treatment that we propose to implement in our study. The objective here is to first present a brief review of the topic. Due to poor evidence in the field, we propose a pilot study to evaluate the effect of a mandibular advancement device in patients with cardiovascular disease who are not treated for their sleep pathology in order to improve their therapeutic management.

## 1. Justification

Obstructive sleep apnea (OSA) leads to hypoxemia, sleep fragmentation with excessive daytime sleepiness, reduced quality of life, and altered neurocognitive performance [1]. In the HypnoLaus study in 2015, the prevalence of moderate to severe sleep-disordered breathing (≥15 events per h) in the general population was 23.4% in women and 49.7% in men [2]. The review published by Senaratna et al. in 2017 established a prevalence between 9 and 38% for an Apnea Hypopnea Index (AHI) > 5 and between 6 and 17% for an AHI > 15/hour in the general population [3]. Advanced age, male sex, and a high BMI increased the prevalence. OSA is a worldwide public health problem because of the potentially fatal consequences it can generate (traffic accidents and stroke). Approximately 80% of apneic patients remain undiagnosed [4].

The prevalence of OSA in cardiac patients is higher than the prevalence observed in the general population.

Several studies have shown that OSA is a cardiovascular (CV) risk factor and is associated with cardiovascular events (ventricular hypertrophy, hypertension, endothelial dysfunction, and mortality) [5]. In patients with cardiovascular diseases (CVD), a prevalence of 40 to 80% depending on the cardiac pathology studied is observed [6], and in patients with coronary disease, OSA is a common condition with a prevalence of 40% to 60% [7,8].

Continuous Positive Airway Pressure (CPAP) is and remains the gold standard therapy for moderate to severe OSA [9]. However, CPAP has its limitations: according to the author, “clinical studies show that less than 50% of patients use it for more than 4 h per night” and therefore will not wear CPAP enough for the treatment to be beneficial [10].

However, the large trials, including SAVE (Sleep Apnea cardiovascular Endpoints), failed to prove the benefit of CPAP in the reduction of cardiovascular events. Indeed the SAVE study [8] enrolled adults with moderate to severe OSA and coronary artery disease or cerebrovascular disease that were enrolled in order to identify second cardiovascular events in this population.

Results highlight that CPAP did not prevent cardiovascular events but improved health-related quality of life [11]. However, this impact is greatly limited by poor patient compliance with significant rate-stop device treatment use [12,13]. The mean adherence decreased rapidly and was only 3.3 h per night during follow-up. Importantly, this poor adherence has been presented as the main reason for the negative result, as the impact of CPAP depends on the duration of wearing it but also on the importance of wearing it at the end of the night (during the period of rapid eye movement with the strongest apneas) [14]. Poor adherence has been frequently reported with CPAP in those with appropriate indications for its use [15]. More importantly, in patients with CVD, who present more often with few symptoms, adherence could be even worse [16]. This necessitates an alternative in order to improve daytime sleepiness and quality of life.

Mandibular advancement devices (MAD) have emerged as the leading alternative to CPAP. MAD is a noninvasive and appropriate treatment for patients who are intolerant of CPAP and may be comparable to CPAP in mild disease [17].

Oral appliances (OA) protrude the mandible during sleep to maintain upper airway patency and include a titratable mechanism that allows for a gradual protrusion. Their principle is to increase and stabilize the oropharyngeal and hypopharyngeal airway space by repositioning and maintaining the lower jaw in a forward position during sleep [18,19].

With MAD, the upper airway occurs in both the lateral and the anteroposterior dimension due to the anterior displacement of the base of the tongue, epiglottis, and soft palate with OA [13,20].

Various authors propose recommendations to use the MAD in patients with mild to moderate OSA as a therapeutic alternative following a failure or refusal of CPAP for severe OSA. The MAD treatment follows a dental check-up and is carried out by dentists with training in sleep medicine [21,22].

A number of trials have shown comparable effects of MAD and CPAP in OSA patients with regard to symptoms such as daytime sleepiness. Recently, studies revealed similar beneficial changes in cardiac autonomic function during the day, especially in blood pressure [23] and reverse left ventricular hypertrophic remodeling (refer to Table 1). CPAP remains more effective than MAD in eliminating hypoxic events [24,25]. To our knowledge, no study has shown clinical benefits of MAD on CV events in any clinical setting. Furthermore, the effect of MAD in a cohort of coronary patients with OSA on cardiovascular events has yet to be reported.

A randomized controlled trial concluded that CPAP and MAD may have similar effectiveness in reducing the risk of fatal cardiovascular events in patients with severe OSA [31]. Several studies have concluded that MADs are a good alternative to CPAP because of their comfort, ease of use, and lower cost. These different parameters could explain the better compliance of patients [32].

In 2021, a study by Xu L. et al. found no significant difference in the average hours of use between CPAP and MAD [33]. In 2020, the Uniken Venema team published a study on the long-term follow-up of the therapeutic management of OSA. After 10 years, patients wore MAD for an average of 7.8 h/night, compared to 6.8 h/night for CPAP [29].

CPAP and MAD also demonstrate an effect on other criteria, including daytime sleepiness, quality of life, and blood pressure [17]. MAD is an alternative therapy recommended for patients who refuse or cannot tolerate CPAP. Patients are more tolerant and compliant with long-term MAD therapy [34].

However, there are some contraindications to the placement of MAD, and the patient’s oral condition will determine the feasibility of MAD. Indeed, teeth that are too dilapidated will represent a temporary contraindication to the placement of MAD. If more care is required, the feasibility will then be re-evaluated.

The ideal dental conditions for the placement are: healthy, retentive teeth (the orthosis clips onto the teeth), non-mobile, with a healthy periodontal support, all the teeth present or a small number of missing teeth, a mouth opening greater than 35 mm, a mandibular propulsion of at least six mm, an absence of musculo-articular pathology, and an absence of gag reflex.

Periodontal disease may increase the risk of cardiovascular disease. Periodontal disease can cause tooth mobility due to loss of bone support and tooth loss, which can even contraindicate the placement of MAD [35].

Periodontal diseases, infectious pathologies of the supporting tissues of the teeth, lead to gingival inflammation, irreversible bone loss, and therefore mobility of the teeth. This pathology is more frequent in cardiac patients and may therefore represent a contraindication to the establishment of MADs.

When we searched the keywords “sleep apnea syndromes” and “oral appliance”, currently on https://www.clinicaltrials.gov (accessed on 17 June 2022) 90 studies appear (85 MAD and OSA).

Of these 90 studies, only 3 relate more specifically to the cardiovascular field and focus on the influence on the primary prevention of an ischemic stroke, on the effect of atrial fibrillation, and on the impact of MAD on cardiac remodeling. This last study seems to be the most relevant because it is a randomized interventional study.

To date, there are no studies on mandibular advancement orthoses and periodontitis, or on other cardiac conditions and sleep apnea, which explains our interest in this subject.

## 2. Study Design

The aim of our work is to assess the oral status of patients with OSA at risk of developing heart disease. This oral condition will then make it possible to validate the feasibility of an orthosis in patients who have failed or refused CPAP treatment.

We propose a study assessing the management by MAD in a cohort of patients with CVD, proven OSA, and CPAP failure. A first oral examination prior to any inclusion will determine the existence of periodontal disease, given the major correlations between periodontal disease and cardiovascular risk. Indeed, in this population, the prevalence of periodontitis in patients with OSA “was 77–79% depending on the definition used” which is almost 4 times higher than the general adult population [36,37]. This epidemiological link is supported by reliable biological evidence, showing that periodontal disease may unfavorably modulate cardiovascular risk, whereby patients with periodontitis have an increased frequency of overweight, hypertension, endothelial dysfunction, and dyslipidemia [38]. Consistently, a recent consensus report by Sanz highlights the need for regular follow-up at least once per year [37]. Patients with unstable periodontal disease cannot be included in this study but will be referred for periodontal management.

During the inclusion phase, a detailed oral examination (dental, periodontal, mucous membranes, muscular, and articular) and a dedicated complementary exam (panoramic radiograph and teleradiography) will be carried out in order to validate the feasibility of this management by MAD on stabilized periodontal terrain. This study will allow us to determine specific predictive factors for the effectiveness of MAD and identify characteristics of patients who respond well to MAD therapy. In addition, quality of life will be evaluated by self-questionnaires and an analysis of cardiac monitoring parameters (systolic pressure, etc.). After MAD placement, regular follow-up is necessary two times per year in order to check the oral status of patients and record the occurrence of adverse effects of MAD.

This study demonstrates the importance of and may make possible multidisciplinary care (cardiologists, sleep specialists, internists, and odontologists) for people with OSA:

On the one hand, there is OSA, the diagnosis of which is still a challenge in this population.

On the other hand, to show the importance of restoring oral health in this at-risk population for maintaining overall health [39].

## 3. How to Improve the Management of OSA?

The role of the dentist in screening for OSA: he sees a large number of patients every day and plays an important role in screening for this underdiagnosed pathology. A questionnaire given in the waiting room, the questioning at the beginning of the consultation, the exobuccal examination, and the intraoral examination can make it possible to suspect OSA, and thus the practitioner can refer the patient to a specialized doctor.

The non-management of OSA following a failure or refusal of CPAP represents a real public health problem because of the potentially serious risks of associated comorbidities.

It would be necessary to be able to inform and sensitize the patients to the alternative to CPAP, which is the MAD.

The sleep doctor must be able to guide his patients in the event of treatment failure or refusal.

Reminder/table with items to check:

- Patient failing to comply with or refuses CPAP
- MAD can be proposed as secondary therapy
- Feasibility conditions: elements to observe
  ○ good dental condition (dental and/or periodontal care to be carried out upstream then reassessment of feasibility),
  ○ absence of infectious foci
  ○ sufficient number of teeth
  ○ no tooth mobility
  ○ mouth opening greater than 40 mm
  ○ mandibular propulsion of at least 5 mm
  ○ no psychiatric illness
  ○ no progressive temporomandibular dysfunctions
  ○ no significant gag reflexes
- The sleep doctor informs the patient of the modalities of this therapeutic alternative.
- Placement of the MAD and regular follow-up with a dental surgeon

## Figures and Tables

**Table 1:** Studies and effectiveness of MADs on the general population between 2014 and 2022.

Study	Sample Size	Age	AHI Baseline	AHI after Therapy	Follow-Up	Outcomes Reported
Dal Fabbro 2014 [26]	29	47 ± 8.9	42.3 ± 24.3	25 ± 12.4	1 month	PSG, ESS, ABPM, OS, HRV
Glos 2016 [24]	40	49.5 ± 11.8	28.5 ± 16.5	<10	12 weeks	PSG, HRV, BP, ESS
Gagnadoux 2017 [27]	150	53.8 ± 10.2	40 (34 to 50.5)		2 months	PSG, BP, EF, ESS
	75 effective MAD			18.5 (11.5 to 26)		
	75 sham device			38 (23 to 51)		
Barbosa 2020 [28]	20	51.91 ± 12.66	27.15	6.16	3 months	PSG, ESS
Uniken Venema 2020 [29]	14	61	31.7 ± 20.6	9.9 ± 10.3	10 years	PSG
Dieltjens 2022 [25]	63	49 ± 11	11.7 (8.2 to 24.9)	NC	6 months	PSG, BP, LV function
Belkhode 2022 [30]	40	30 to 50			3 months	PSG, ESS

Abbreviations: AHI: Apnea Hypopnea Index, MAD: mandibular advancement device, ESS: Epworth sleepiness scale, PSG: full night PolySomnography, ABPM: 24-h ambulatory blood pressure monitoring, OS: blood samples analyses for oxidative stress parameters, HRV: heart rate variability, BP: blood pressure, EF: endothelial function, FOSQ: functional outcomes of sleep questionnaire, LV: left ventricular, NC: not communicated.

## Data Availability

Not applicable.
